# Multi-Responsive Silk Fibroin-Based Nanoparticles for Drug Delivery

**DOI:** 10.3389/fchem.2020.585077

**Published:** 2020-11-03

**Authors:** Ya Ma, Brandon S. B. Canup, Xiaoling Tong, Fangyin Dai, Bo Xiao

**Affiliations:** ^1^State Key Laboratory of Silkworm Genome Biology, Key Laboratory of Sericultural Biology and Genetic Breeding, Ministry of Agriculture and Rural Affairs, College of Sericulture, Textile and Biomass Sciences, Southwest University, Chongqing, China; ^2^Chongqing Key Laboratory of Soft-Matter Material Chemistry and Function Manufacturing, School of Materials and Energy, Southwest University, Chongqing, China; ^3^Department of Chemistry, Georgia State University, Atlanta, GA, United States

**Keywords:** multi-responsibility, drug delivery, silk fibroin, nanoparticle, β-sheet

## Abstract

Silk fibroin has the merits of biocompatibility, biodegradability, ease of processing, and feasibility of modification, which present it as a promising drug delivery material. This review focuses on the structures of silk fibroin, the controlled transformation of secondary structures, and the formation mechanism of silk fibroin-based nanoparticles (SFNPs). We also discuss the intrinsic multi-responsive, surface functionalization, and transgenic modification of SFNPs for drug delivery.

## Introduction

Drug delivery is required to deliver appropriate amounts of therapeutic agents to the diseased sites to improve the therapeutic effect of drugs and reduce their adverse effects (Tian et al., 2014; Jain, 2020). To achieve these goals, various nanoscale drug delivery systems, including mesoporous silica nanoparticles (NPs), liposomes, and polymeric NPs, have been developed in recent years (Chen H. et al., 2018; Patra et al., 2018). Among them, polymeric NPs have attracted increasing attention due to their numerous advantageous features such as good biocompatibility, desirable biodegradability, and ease of functionalization (Sundar et al., 2010; Merkle, 2015).

In contrast to traditional polymeric NPs, polymeric drug carriers that respond to the external stimuli (e.g., pH, ROS, GSH, enzyme, temperature, and light) by changing their physicochemical properties can maintain the stability of the loaded drugs, prolong the blood circulation time of drugs, realize on-demand drug release in the targeted cells, and reduce the systemic side toxicities (Cheng et al., 2014; Guragain et al., 2015; Fu et al., 2018; Gao et al., 2019). However, many of these stimuli-responsive polymers are synthesized through the integration of multiple functional chemical groups via complex chemical reactions, which involve large amounts of organic solvents and harsh reaction environments, eventually resulting in potential toxicity and high expense (Lei et al., 2017; Bordat et al., 2019; Deng et al., 2020). In recent years, a number of natural polymers, including chitosan, alginate, gelatin, and silk fibroin, have been developed as drug delivery materials. Among them, silk fibroin is an FDA-approved polymer that can be processed into nanoscale particles in the mild environment (Lammel et al., 2011; Kundu et al., 2014; George et al., 2019). For instance, ionic liquid-silk fibroin solutions were prepared and used to fabricate SFNPs under ultrasounds (Lozano-Pérez et al., 2014). The silk fibroin-based NPs (SFNPs) not only have the merits of excellent biocompatibility and desirable biodegradability, but also show the features of multi-responsive (Maitz et al., 2017; Wongpinyochit et al., 2018; Gou et al., 2019b). In addition, they can efficiently load small-molecule drugs, proteins, and nucleic acids through surface adsorption, physical encapsulation, and chemical coupling, which are able to prevent drug degradation, optimize the drug pharmacokinetics, and increase the cellular uptake amounts of drugs (Zhao et al., 2015).

In this review, we summarize the controlled transformation of the secondary structures, the multiple stimuli-responsive capacities, and the surface/multifunctional modification of SFNPs for drug delivery.

## Structure of Silk Fibroin

### Primary Structure of Silk Fibroin

Silk fibroin consists of 18 kinds of amino acids, in which Gly is the most abundant amino acid accounting for 43% of all amino acids, followed by Ala (29%) and Ser (12%) (Qi et al., 2017). Silk fibroin is composed of three basic subunits, a heavy chain (H-chain), a light chain (L-chain), and a P25 gene-encoded glycoprotein, whose ratio is 6:6:1. In particular, the H-chain (~350 kDa) has 12 hydrophobic blocks and 11 hydrophilic blocks, which is the main contributor of β-sheet structures in SFNPs. These hydrophobic β-sheet blocks are constituted of the repeat sequence GAGAGS and are formed on the basis of intramolecular and intermolecular hydrogen bonds (mainly between Gly and Ala), van der Waals force, and hydrophobic interaction, which confer SFNPs with stable 3-dimensional structures (Nguyen et al., 2019; Montalbán et al., 2020; Pham and Tiyaboonchai, 2020). In the context of the L-chain (26 kDa), its primary structure has no amino acid repeat sequence, and it conjugates with the H-chain through a disulfide bond. The main function of the L-chain is to assist with the secretion of the H-chain from the silk gland of silkworm. In addition, the bio-function of P25 glycoprotein (30 kDa) is similar to that of the L-chain.

### Secondary Structure of Silk Fibroin

Silk fibroin has two main types of crystal structures, namely Silk I and Silk II. Silk I is a transition state, which contains random coils, α-helical structures, and other amorphous structures. Silk II is composed of antiparallel β-sheet crystal structures, which make silk fibroin insoluble in aqueous solutions (Cebe et al., 2017). In nature, Silk I exists in the silk gland, while Silk II exists in the form of spun silk fiber. Thus, the investigation of the silk spinning mechanism can uncover the influencing factors in the structure transformation process of silk fibroin (Li et al., 2015; Pham et al., 2018b). This information can then be utilized for understanding the formation mechanism of SFNPs.

The formation of SFNPs is based on the structure transformation from Silk I (random coil and α-helical structure) to Silk II (highly ordered β-sheet). Silk fibroin molecules form loose amorphous structures in aqueous solution due to their intrinsic electrostatic repulsion (Zhang et al., 2006). Meanwhile, water tends to couple with these silk fibroin molecules and form a layer of hydration film. Upon the external treatment, β-sheet structures are formed, resulting in the self-assembly of molecular chains and the formation of SFNPs (Mottaghitalab et al., 2015; Zhao et al., 2015).

The self-assembly method has been commonly used to produce SFNPs. It is known that self-assembly, a thermodynamic process, is determined by molecular aggregation, which can be modulated by external environmental factors (Lu et al., 2012; Bai et al., 2013). Under certain external stimulation such as metal ion, low temperature, organic solvent, and ultrasound, the soluble, and irregular Silk I can be transformed into non-soluble Silk II (Hu et al., 2011; Terada et al., 2016). On the other hand, under high concentration of neutral salt and other certain conditions (e.g., acid, ROS, enzyme, and hyperthermia), the β-sheet structures of Silk II undergo a conformational reversion to amorphous structures of Silk I (Wongpinyochit et al., 2018). Therefore, the transformation between the crystal structures of silk fibroin is a complex and multi-factorial regulated process, which is fundamental to the multi-responsive property of SFNPs.

## Multi-Stimuli-Responsive of SFNPs

**Figure 1:**
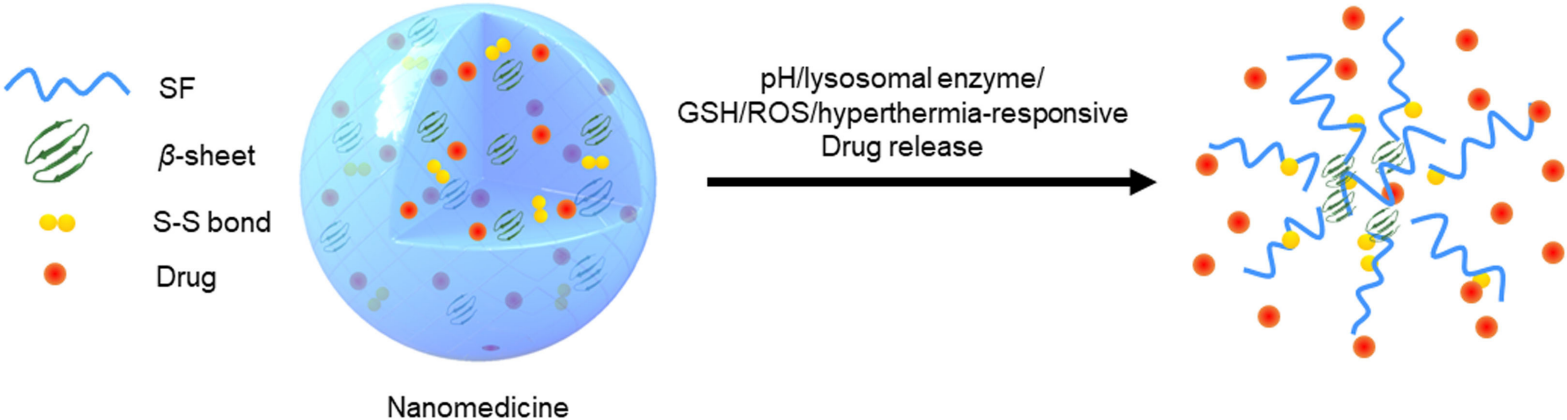
Schematic illustration of multi-responsive properties of SFNPs.

Nanotherapeutics with multi-responsive can achieve spatial and temporal release of drugs in diseased tissues (Qu et al., 2018). Kaplan group was the first to report that SFNPs showed an obvious pH-dependent drug release property. The release rate of doxorubicin (DOX) from SFNPs was significantly increased in the buffer (pH 4.5) in comparison with that in the buffers with the pH values of 7.4 and 6.0. They speculated that the loss of the negative net charges in the buffer (pH 4.5) weakened the electrostatic interaction between silk fibroin molecules and DOX, resulting in the accelerated release of DOX from NPs (Seib et al., 2013). In addition, *Totten et al.*,. studied the DOX release behaviors of PEGylated SFNPs in the acidic buffers with or without lysosomal enzymes. They found that the DOX release rate was significantly increased in the simulated lysosomal fluid (lysosomal enzyme and acidic environment), providing direct evidence of the accelerated release of DOX in the lysosome of tumor cells (Totten et al., 2017). Very recently, our group not only confirmed the pH responsive of SFNPs, but also discovered that they had obvious ROS/GSH/hyperthermia-responsive properties, as shown in Figure 1. We further discovered a potential mechanism for their pH/ROS/GSH/hyperthermia-responsive properties. Protons, H_2_O_2_ molecules, and hyperthermia could gradually destroy the hydrogen bonds in β-sheet structures, and GSH could reduce the internal disulfide bonds into sulfhydryl groups. The treatments with the protons, H_2_O_2_ molecules, hyperthermia, and GSH loosen the compact structures of SFNPs leading to the acceleration of drug release from these NPs (Gou et al., 2019a, 2020). These results collectively reveal that SFNPs have obvious pH/ROS/GSH/hyperthermia/lysosomal enzyme-responsive properties, which can facilitate the specific drug release in the targeted cells via microenvironmental stimuli.

## Modification of SFNPs

Many chemically active groups such as amino groups, carboxyl groups, and sulfhydryl groups, are present in the backbone of silk fibroin, and these groups are able to be used for chemical modifications, which can endow SFNPs with some advanced functions (e.g., charge reversal, controlled drug release, and targeting property) (Wenk et al., 2011; Chen J. et al., 2018; Pandey et al., 2020).

For instance, to improve the tumor-targeting property, Sun et al. prepared DOX-loaded SFNPs and further functionalized their surface with folic acid (FA) through the chemical reaction between the amino groups of silk fibroin and the carboxyl groups of FA molecules. The obtained FA-DOX-NPs could be specifically internalized by FA receptor-overexpressed tumor cells and release the loaded DOX in a controlled manner. They further found that the surface functionalization of FA significantly improved the chemotherapeutic effect and reduced the potential adverse effects of DOX (Sun et al., 2018). Recently, Pham et al. fabricated a new type of SFNPs by using a reactive carbodiimide (EDC) or polyethyleneimine (PEI). It was found that the hydrodynamic particle sizes of all the developed cross-linked NPs were similar to the traditional SFNPs and their zeta potentials were controllably altered from a negative charge to positive. In addition, the crystallinity of these NPs increased with increasing the amount of EDC or decreasing the PEI content, which can improve drug encapsulation efficiency (Pham et al., 2018a).

Transgenic technology is another promising strategy to produce the modified silk fibroin by inserting or replacing genes in the silkworm genome to generate novel silk fibroin derivatives (Shi et al., 2014; Helfricht et al., 2016). This strategy has attracted increasing attention in recent years, as it can fundamentally alter the primary structure of silk fibroin. Xia et al. adjusted the proportion of elastin in silk fibroin using genetic engineering technology and obtained silk-elastin like proteins (SELPs), which could form NPs *via* self-assembly. The first step was the spontaneous formation of micelles with silk blocks as the core structures, which was driven by hydrogen bonds among silk blocks; the second step was driven by the hydrophobic interactions among elastin blocks, leading to the orderly association of SELP molecules. During the assembly processes, drugs could be encapsulated in the SELP matrix to form NPs (Xia et al., 2011).

## Conclusions

Silk fibroin has become an attractive natural polymer for drug delivery due to its versatile merits such as good biocompatibility, modulated biodegradability, large scale production, easy modification, and self-assembling property. Many approaches can be applied to further the application of silk fibroin as a drug delivery material such as optimization of its primary structures, modification with functional chemical groups, adjusting the self-assembling processes, and controlling the interaction between silk fibroin and the loaded agents. It is also critical to endow them with a diseased site-targeting capacity *via* the conjugation of targeting ligands. Furthermore, the alteration of the contents of hydrophobic β-sheet structures and disulfide bonds is important to improve the responsive capacities of these silk fibroin-based NPs (SFNPs) to acidity, ROS, and GSH, which can facilitate the on-demand release of the loaded drugs from the NPs. Collectively, these SFNPs can be exploited as a promising nanocarrier for drug delivery.

## Author Contributions

YM wrote the draft of the manuscript. BC, XT, FD, and BX provided suggestions and edited the manuscript. All authors contributed to the article and approved the submitted version.

## Conflict of Interest

The authors declare that the research was conducted in the absence of any commercial or financial relationships that could be construed as a potential conflict of interest.
